# 4-Meth­oxy-*N*-(1-methyl-1*H*-indazol-5-yl)benzene­sulfonamide

**DOI:** 10.1107/S1600536813021624

**Published:** 2013-08-07

**Authors:** Hakima Chicha, El Mostapha Rakib, Detlef Geffken, Mohamed Saadi, Lahcen El Ammari

**Affiliations:** a Laboratoire de Chimie Organique et Analytique, Université Sultan Moulay Slimane, Faculté des Sciences et Techniques, Béni-Mellal, BP 523, Morocco; b Department of Pharmaceutical Chemistry, Institute of Pharmacy, University of Hamburg, Hamburg, Germany; c Laboratoire de Chimie du Solide Appliquée, Faculté des Sciences, Université Mohammed V-Agdal, Avenue Ibn Battouta, BP 1014, Rabat, Morocco

## Abstract

The indazole ring system [maximum deviation = 0.013 (2) Å] of the title compound, C_15_H_15_N_3_O_3_S, makes a dihedral angle of 50.11 (7)° with the benzene ring. In the crystal, cohesion is provided by C—H⋯O and N—H⋯N hydrogen bonds, which link the molecules into chains propagating along the *b-*axis direction.

## Related literature

For the pharmacological activity of sulfonamide derivatives, see: Bouissane *et al.* (2006 ▶); El-Sayed *et al.* (2011 ▶); Mustafa *et al.* (2012 ▶). For similar compounds, see: Abbassi *et al.* (2012 ▶, 2013 ▶); Chicha *et al.* (2013 ▶).
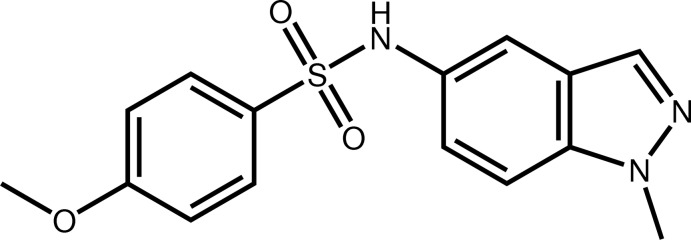


## Experimental

### 

#### Crystal data

C_15_H_15_N_3_O_3_S
*M* *_r_* = 317.36
Monoclinic, 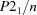

*a* = 10.1069 (3) Å
*b* = 13.6178 (3) Å
*c* = 10.8530 (2) Å
β = 90.777 (2)°
*V* = 1493.60 (6) Å^3^
*Z* = 4
Mo *K*α radiation
μ = 0.23 mm^−1^
*T* = 296 K
0.39 × 0.33 × 0.23 mm

#### Data collection

Bruker X8 APEXII diffractometer
Absorption correction: multi-scan (*SADABS*; Bruker, 2009 ▶) *T*_min_ = 0.651, *T*_max_ = 0.747
19007 measured reflections
4178 independent reflections
3232 reflections with *I* > 2σ(*I*)
*R*_int_ = 0.029

#### Refinement

*R*[*F* ^2^ > 2σ(*F* ^2^)] = 0.041
*wR*(*F* ^2^) = 0.125
*S* = 1.02
4178 reflections
199 parameters
H-atom parameters constrained
Δρ_max_ = 0.29 e Å^−3^
Δρ_min_ = −0.28 e Å^−3^

### 

Data collection: *APEX2* (Bruker, 2009 ▶); cell refinement: *SAINT* (Bruker, 2009 ▶); data reduction: *SAINT*; program(s) used to solve structure: *SHELXS97* (Sheldrick, 2008 ▶); program(s) used to refine structure: *SHELXL97* (Sheldrick, 2008 ▶); molecular graphics: *ORTEP-3 for Windows* (Farrugia, 2012 ▶); software used to prepare material for publication: *PLATON* (Spek, 2009 ▶) and *publCIF* (Westrip, 2010 ▶).

## Supplementary Material

Crystal structure: contains datablock(s) I. DOI: 10.1107/S1600536813021624/bt6924sup1.cif

Structure factors: contains datablock(s) I. DOI: 10.1107/S1600536813021624/bt6924Isup2.hkl

Supplementary material file. DOI: 10.1107/S1600536813021624/bt6924Isup3.cml

Additional supplementary materials:  crystallographic information; 3D view; checkCIF report

## Figures and Tables

**Table 1:** Hydrogen-bond geometry (Å, °)

*D*—H⋯*A*	*D*—H	H⋯*A*	*D*⋯*A*	*D*—H⋯*A*
N1—H1⋯N2^i^	0.89	2.25	3.1335 (19)	176
C8—H8*A*⋯O1^ii^	0.96	2.49	3.391 (2)	157

Symmetry codes: (i) 
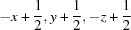
; (ii) 
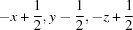
.

**Fig. 1.**
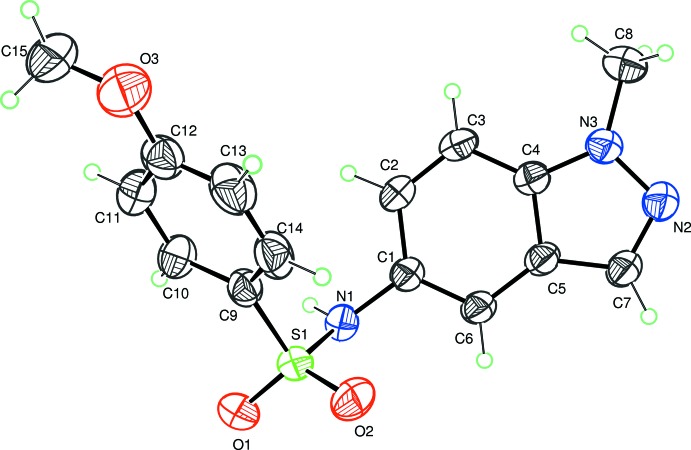
Molecular structure of the title compound with the atom-labelling scheme. Displacement ellipsoids are drawn at the 50% probability level. H atoms are represented as small circles.

**Fig. 2.**
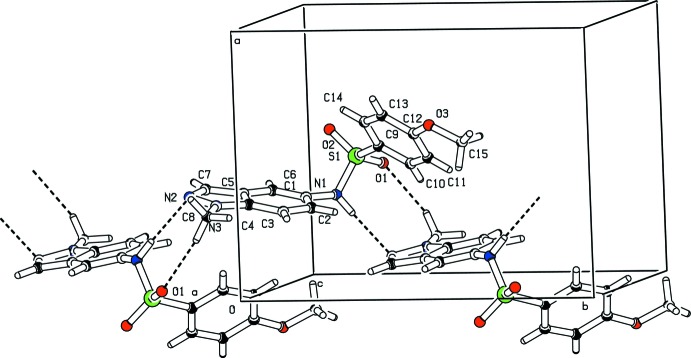
Partial crystal packing for the title compound showing C8–H8A···O1 and N1–H1···N2 hydrogen bonds as dashed lines.

## supplementary crystallographic information

### 1. Comment

Sulfonamide derivatives are well known pharmaceutical agents since this group has been the main functional part of the most of the drug structures due to stability and tolerance in human beings. These compounds exhibit a wide range of biological activities such as anticancer, anti-inflammatory, and antiviral functions (Abbassi *et al.*, 2012; Bouissane *et al.*, 2006; El-Sayed *et al.*, 2011; Mustafa *et al.*, 2012). The present work is a continuation of the investigation of the sulfonamide derivatives published recently by our team (Abbassi *et al.*, 2013; Chicha *et al.*, 2013).

The molecule of 4-Methoxy-*N*-(1-methyl-1*H*-indazol-5-yl)-benzenesulfonamide is built up from the fused five- and six-membered rings (N2 N3 C1—C7) linked to the benzenesulfonamide group as shown in Fig. 1. The fused rings system is planar, with the maximum deviation of -0.013 (2) Å for N2 atom. Moreover, the dihedral angle between the indazole system and the plan through the atoms forming the benzene ring (C9—C14) is of 50.11 (7)°.

In the crystal, the molecules are interconnected by C8–H8A···O1 and N1–H1···N2 hydrogen bonds forming a one-dimensional chain running along the *b* axis as shown in Fig.2 and Table 2.

### 2. Experimental

A mixture of 1-methyl-5-nitroindazole (1.22 mmol) and anhydrous SnCl_2_ (1.1 g, 6.1 mmol) in 25 ml of absolute ethanol was heated at 333 K for 6 h. After reduction, the starting material disappeared, and the solution was allowed to cool down. The pH was made slightly basic (pH 7–8) by addition of 5% aqueous potassium bicarbonate before extraction with ethyl acetate. The organic phase was washed with brine and dried over magnesium sulfate. The solvent was removed to afford the amine, which was immediately dissolved in pyridine (5 ml) and then reacted with 4-methoxybenzenesulfonyl chloride (1.25 mmol) at room temperature for 24 h. After the reaction mixture was concentrated *in vacuo*, the resulting residue was purified by flash chromatography (eluted with Ethyl acetate: Hexane 1:9). The title compound was recrystallized from acetone.

### 3. Refinement

H atoms were located in a difference map and treated as riding with C–H = 0.96 Å, C–H = 0.93 Å, and N–H = 0.89 Å for methyl, aromatic CH and NH respectively. All hydrogen with *U*_iso_(H) = 1.2 *U*_eq_ (aromatic, NH) and *U*_iso_(H) = 1.5 *U*_eq_ for methyl.

### Crystal data

**Table:**

C_15_H_15_N_3_O_3_S	*F*(000) = 664
*M**_r_* = 317.36	*D*_x_ = 1.411 Mg m^−^^3^
Monoclinic, *P*2_1_/*n*	Mo *K*α radiation, λ = 0.71073 Å
Hall symbol: -P 2yn	Cell parameters from 4178 reflections
*a* = 10.1069 (3) Å	θ = 2.4–29.6°
*b* = 13.6178 (3) Å	µ = 0.23 mm^−^^1^
*c* = 10.8530 (2) Å	*T* = 296 K
β = 90.777 (2)°	Block, colourless
*V* = 1493.60 (6) Å^3^	0.39 × 0.33 × 0.23 mm
*Z* = 4	

### Data collection

**Table:**

Bruker X8 APEXII diffractometer	4178 independent reflections
Radiation source: fine-focus sealed tube	3232 reflections with *I* > 2σ(*I*)
Graphite monochromator	*R*_int_ = 0.029
φ and ω scans	θ_max_ = 29.6°, θ_min_ = 2.4°
Absorption correction: multi-scan (*SADABS*; Bruker, 2009)	*h* = −14→13
*T*_min_ = 0.651, *T*_max_ = 0.747	*k* = −18→18
19007 measured reflections	*l* = −15→15

### Refinement

**Table:**

Refinement on *F*^2^	Primary atom site location: structure-invariant direct methods
Least-squares matrix: full	Secondary atom site location: difference Fourier map
*R*[*F*^2^ > 2σ(*F*^2^)] = 0.041	Hydrogen site location: inferred from neighbouring sites
*wR*(*F*^2^) = 0.125	H-atom parameters constrained
*S* = 1.02	*w* = 1/[σ^2^(*F*_o_^2^) + (0.0694*P*)^2^ + 0.2788*P*] where *P* = (*F*_o_^2^ + 2*F*_c_^2^)/3
4178 reflections	(Δ/σ)_max_ < 0.001
199 parameters	Δρ_max_ = 0.29 e Å^−^^3^
0 restraints	Δρ_min_ = −0.28 e Å^−^^3^

### Special details

**Table:**

Geometry. All s.u.'s (except the s.u. in the dihedral angle between two l.s. planes) are estimated using the full covariance matrix. The cell s.u.'s are taken into account individually in the estimation of s.u.'s in distances, angles and torsion angles; correlations between s.u.'s in cell parameters are only used when they are defined by crystal symmetry. An approximate (isotropic) treatment of cell s.u.'s is used for estimating s.u.'s involving l.s. planes.
Refinement. Refinement of *F*^2^ against all reflections. The weighted *R*-factor *wR* and goodness of fit *S* are based on *F*^2^, conventional *R*-factors *R* are based on *F*, with *F* set to zero for negative *F*^2^. The threshold expression of *F*^2^ > 2σ(*F*^2^) is used only for calculating *R*-factors(gt) *etc*. and is not relevant to the choice of reflections for refinement. *R*-factors based on *F*^2^ are statistically about twice as large as those based on *F*, and *R*- factors based on all data will be even larger.

### Fractional atomic coordinates and isotropic or equivalent isotropic displacement parameters (Å^2^)

**Table:**

	*x*	*y*	*z*	*U*_iso_*/*U*_eq_	
C1	0.35240 (14)	0.12964 (10)	0.31509 (12)	0.0369 (3)	
C2	0.32504 (16)	0.16841 (11)	0.19599 (14)	0.0411 (3)	
H2	0.3091	0.2353	0.1875	0.049*	
C3	0.32149 (15)	0.11009 (11)	0.09337 (13)	0.0395 (3)	
H3	0.3025	0.1356	0.0156	0.047*	
C4	0.34782 (13)	0.01018 (10)	0.11104 (12)	0.0341 (3)	
C5	0.37682 (14)	−0.02927 (10)	0.22857 (13)	0.0364 (3)	
C6	0.37813 (15)	0.03159 (10)	0.33265 (13)	0.0379 (3)	
H6	0.3958	0.0065	0.4108	0.045*	
C7	0.39589 (17)	−0.13088 (11)	0.20596 (14)	0.0452 (4)	
H7	0.4174	−0.1766	0.2666	0.054*	
C8	0.32417 (16)	−0.06444 (13)	−0.10173 (13)	0.0479 (4)	
H8A	0.2402	−0.0949	−0.1185	0.072*	
H8B	0.3925	−0.0996	−0.1437	0.072*	
H8C	0.3221	0.0023	−0.1301	0.072*	
C9	0.53540 (14)	0.33142 (10)	0.36119 (13)	0.0379 (3)	
C10	0.46991 (15)	0.42076 (11)	0.35614 (15)	0.0437 (3)	
H10	0.4051	0.4347	0.4136	0.052*	
C11	0.49956 (17)	0.48930 (11)	0.26688 (15)	0.0472 (4)	
H11	0.4541	0.5486	0.2632	0.057*	
C12	0.59753 (18)	0.46877 (13)	0.18314 (15)	0.0494 (4)	
C13	0.6648 (2)	0.37995 (15)	0.18895 (16)	0.0586 (5)	
H13	0.7313	0.3667	0.1329	0.070*	
C14	0.63377 (17)	0.31130 (12)	0.27713 (15)	0.0498 (4)	
H14	0.6786	0.2517	0.2803	0.060*	
C15	0.5734 (3)	0.62492 (16)	0.0850 (2)	0.0806 (7)	
H15A	0.6100	0.6615	0.0179	0.121*	
H15B	0.5874	0.6603	0.1605	0.121*	
H15C	0.4802	0.6159	0.0709	0.121*	
N1	0.35058 (13)	0.19331 (9)	0.42115 (11)	0.0409 (3)	
H1	0.2838	0.2355	0.4224	0.049*	
N2	0.37938 (15)	−0.15242 (9)	0.08822 (12)	0.0469 (3)	
N3	0.35107 (12)	−0.06604 (9)	0.03012 (11)	0.0395 (3)	
O1	0.45149 (14)	0.29493 (9)	0.58123 (10)	0.0567 (3)	
O2	0.58740 (13)	0.16972 (9)	0.47644 (12)	0.0579 (3)	
O3	0.63640 (17)	0.53163 (11)	0.09322 (13)	0.0755 (4)	
S1	0.48793 (4)	0.24415 (3)	0.47110 (3)	0.04197 (13)	

### Atomic displacement parameters (Å^2^)

**Table:**

	*U*^11^	*U*^22^	*U*^33^	*U*^12^	*U*^13^	*U*^23^
C1	0.0415 (7)	0.0326 (7)	0.0365 (7)	−0.0015 (5)	−0.0014 (5)	0.0031 (5)
C2	0.0483 (8)	0.0321 (7)	0.0429 (7)	0.0028 (6)	−0.0013 (6)	0.0080 (6)
C3	0.0421 (8)	0.0392 (7)	0.0370 (7)	0.0019 (6)	−0.0040 (6)	0.0099 (6)
C4	0.0306 (6)	0.0362 (7)	0.0356 (6)	−0.0032 (5)	−0.0025 (5)	0.0033 (5)
C5	0.0394 (7)	0.0314 (6)	0.0382 (7)	−0.0036 (5)	−0.0061 (5)	0.0062 (5)
C6	0.0459 (8)	0.0325 (7)	0.0352 (6)	−0.0043 (6)	−0.0044 (5)	0.0061 (5)
C7	0.0576 (10)	0.0321 (7)	0.0455 (8)	−0.0008 (6)	−0.0100 (7)	0.0048 (6)
C8	0.0482 (9)	0.0578 (10)	0.0375 (7)	−0.0042 (7)	−0.0038 (6)	−0.0005 (7)
C9	0.0381 (7)	0.0349 (7)	0.0404 (7)	0.0012 (6)	−0.0068 (5)	−0.0049 (5)
C10	0.0387 (8)	0.0394 (8)	0.0530 (8)	0.0038 (6)	0.0047 (6)	0.0003 (6)
C11	0.0449 (8)	0.0376 (8)	0.0589 (9)	−0.0007 (6)	−0.0006 (7)	0.0026 (7)
C12	0.0552 (10)	0.0494 (9)	0.0438 (8)	−0.0136 (8)	0.0020 (7)	−0.0036 (6)
C13	0.0637 (11)	0.0624 (11)	0.0502 (9)	−0.0011 (9)	0.0174 (8)	−0.0140 (8)
C14	0.0531 (9)	0.0452 (9)	0.0511 (9)	0.0092 (7)	0.0017 (7)	−0.0142 (7)
C15	0.114 (2)	0.0596 (12)	0.0682 (13)	−0.0229 (13)	−0.0051 (12)	0.0189 (10)
N1	0.0482 (7)	0.0341 (6)	0.0403 (6)	0.0021 (5)	0.0011 (5)	−0.0004 (5)
N2	0.0566 (8)	0.0359 (6)	0.0480 (7)	−0.0003 (6)	−0.0074 (6)	−0.0004 (5)
N3	0.0433 (7)	0.0386 (6)	0.0366 (6)	−0.0015 (5)	−0.0047 (5)	0.0004 (5)
O1	0.0813 (9)	0.0532 (7)	0.0355 (5)	0.0041 (6)	−0.0057 (5)	−0.0049 (5)
O2	0.0621 (8)	0.0473 (7)	0.0638 (7)	0.0159 (6)	−0.0164 (6)	0.0051 (6)
O3	0.0955 (11)	0.0686 (9)	0.0631 (8)	−0.0169 (8)	0.0209 (8)	0.0082 (7)
S1	0.0531 (2)	0.0361 (2)	0.03648 (19)	0.00611 (15)	−0.00831 (15)	−0.00026 (13)

### Geometric parameters (Å, º)

**Table:**

C1—C6	1.3731 (19)	C9—S1	1.7553 (15)
C1—C2	1.4199 (19)	C10—C11	1.381 (2)
C1—N1	1.4415 (18)	C10—H10	0.9300
C2—C3	1.368 (2)	C11—C12	1.382 (2)
C2—H2	0.9300	C11—H11	0.9300
C3—C4	1.3990 (19)	C12—O3	1.360 (2)
C3—H3	0.9300	C12—C13	1.388 (3)
C4—N3	1.3603 (18)	C13—C14	1.377 (3)
C4—C5	1.4111 (18)	C13—H13	0.9300
C5—C6	1.401 (2)	C14—H14	0.9300
C5—C7	1.419 (2)	C15—O3	1.423 (3)
C6—H6	0.9300	C15—H15A	0.9600
C7—N2	1.320 (2)	C15—H15B	0.9600
C7—H7	0.9300	C15—H15C	0.9600
C8—N3	1.4530 (19)	N1—S1	1.6370 (14)
C8—H8A	0.9600	N1—H1	0.8867
C8—H8B	0.9600	N2—N3	1.3633 (18)
C8—H8C	0.9600	O1—S1	1.4332 (12)
C9—C10	1.386 (2)	O2—S1	1.4283 (12)
C9—C14	1.386 (2)		
			
C6—C1—C2	121.47 (13)	C10—C11—C12	119.26 (15)
C6—C1—N1	118.59 (12)	C10—C11—H11	120.4
C2—C1—N1	119.92 (12)	C12—C11—H11	120.4
C3—C2—C1	121.86 (13)	O3—C12—C11	124.13 (17)
C3—C2—H2	119.1	O3—C12—C13	115.78 (16)
C1—C2—H2	119.1	C11—C12—C13	120.08 (15)
C2—C3—C4	116.75 (12)	C14—C13—C12	120.46 (15)
C2—C3—H3	121.6	C14—C13—H13	119.8
C4—C3—H3	121.6	C12—C13—H13	119.8
N3—C4—C3	131.28 (13)	C13—C14—C9	119.70 (15)
N3—C4—C5	106.65 (12)	C13—C14—H14	120.2
C3—C4—C5	122.07 (13)	C9—C14—H14	120.2
C6—C5—C4	120.21 (13)	O3—C15—H15A	109.5
C6—C5—C7	135.76 (13)	O3—C15—H15B	109.5
C4—C5—C7	104.01 (12)	H15A—C15—H15B	109.5
C1—C6—C5	117.62 (12)	O3—C15—H15C	109.5
C1—C6—H6	121.2	H15A—C15—H15C	109.5
C5—C6—H6	121.2	H15B—C15—H15C	109.5
N2—C7—C5	111.63 (13)	C1—N1—S1	119.93 (10)
N2—C7—H7	124.2	C1—N1—H1	114.8
C5—C7—H7	124.2	S1—N1—H1	111.3
N3—C8—H8A	109.5	C7—N2—N3	106.21 (12)
N3—C8—H8B	109.5	C4—N3—N2	111.50 (11)
H8A—C8—H8B	109.5	C4—N3—C8	128.21 (13)
N3—C8—H8C	109.5	N2—N3—C8	120.24 (13)
H8A—C8—H8C	109.5	C12—O3—C15	118.25 (16)
H8B—C8—H8C	109.5	O2—S1—O1	119.82 (7)
C10—C9—C14	119.61 (15)	O2—S1—N1	107.85 (7)
C10—C9—S1	119.11 (12)	O1—S1—N1	104.71 (8)
C14—C9—S1	121.24 (12)	O2—S1—C9	108.02 (8)
C11—C10—C9	120.87 (14)	O1—S1—C9	108.41 (7)
C11—C10—H10	119.6	N1—S1—C9	107.44 (6)
C9—C10—H10	119.6		

### Hydrogen-bond geometry (Å, º)

**Table:**

*D*—H···*A*	*D*—H	H···*A*	*D*···*A*	*D*—H···*A*
N1—H1···N2^i^	0.89	2.25	3.1335 (19)	176
C8—H8*A*···O1^ii^	0.96	2.49	3.391 (2)	157

Symmetry codes: (i) −*x*+1/2, *y*+1/2, −*z*+1/2; (ii) −*x*+1/2, *y*−1/2, −*z*+1/2.
